# Reducing severe cutaneous adverse and type B adverse drug reactions using pre‐stored human leukocyte antigen genotypes

**DOI:** 10.1002/clt2.12098

**Published:** 2022-01-14

**Authors:** Kye Hwa Lee, Dong Yoon Kang, Hyun Hwa Kim, Yi Jun Kim, Hyo Jung Kim, Ju Han Kim, Eun Young Song, James Yun, Hye‐Ryun Kang

**Affiliations:** ^1^ Department of Information Medicine Asan Medical Center Seoul South Korea; ^2^ Drug Safety Center Seoul National University Hospital Seoul South Korea; ^3^ Institute of Convergence Medicine Ewha Womans University Mokdong Hospital Seoul South Korea; ^4^ Department of Digital Health Samsung Advanced Institute for Health Science and Technology Sungkyunkwan University Seoul South Korea; ^5^ Seoul National University Biomedical Informatics and Systems Biomedical Informatics Research Center Division of Biomedical Informatics Seoul National University College of Medicine Seoul South Korea; ^6^ Department of Molecular Medicine and Biopharmaceutical Sciences Graduate School of Convergence Science and Technology and College of Medicine Medical Research Center Seoul National University Seoul South Korea; ^7^ Department of Immunology and Rheumatology Nepean Hospital Sydney New South Wales Australia; ^8^ Faculty of Medicine and Health The University of Sydney Sydney New South Wales Australia; ^9^ Institute of Allergy and Clinical Immunology Seoul National University Medical Research Center Seoul National University College of Medicine Seoul South Korea

**Keywords:** adverse drug reaction, human leukocyte antigen, hypersensitivity, pharmacogenomics, preemptive genotyping

## Abstract

**Background:**

Several type B adverse drug reactions (ADRs), especially severe cutaneous adverse reactions (SCARs), are associated with particular human leukocyte antigen (HLA) genotypes. However, pre‐stored HLA information obtained from other clinical workups has not been used to prevent ADRs. We aimed to simulate the preemptive use of pre‐stored HLA information in electronic medical records to evaluate whether this information can prevent ADRs.

**Methods:**

We analyzed the incidence and the risk of ADRs for selected HLA alleles (HLA‐B*57:01, HLA‐B*58:01, HLA‐A*31:01, HLA‐B*15:02, HLA‐B*15:11, HLA‐B*13:01, HLA‐B*59:01, and HLA‐A*32:01) and seven drugs (abacavir, allopurinol, carbamazepine, oxcarbazepine, dapsone, methazolamide, and vancomycin) using pre‐stored HLA information of transplant patients based on the Pharmacogenomics Knowledge Base guidelines and experts' consensus.

**Results:**

Among 11,988 HLA‐tested transplant patients, 4092 (34.1%) had high‐risk HLA alleles, 4583 (38.2%) were prescribed risk drugs, and 580 (4.8%) experienced type B ADRs. Patients with HLA‐B*58:01 had a significantly higher incidence of type B ADR and SCARs associated with allopurinol use than that of patients without HLA‐B*58:01 (17.2% vs. 11.9%, odds ratio [OR] 1.53 [95% confidence interval {CI} 1.09–2.13], *p* = 0.001, 2.3% versus 0.3%, OR 7.13 [95% CI 2.19–22.69], *p* < 0.001). Higher risks of type B ADR and SCARs were observed in patients taking carbamazepine or oxcarbazepine if they had one of HLA‐A*31:01, HLA‐B*15:02, or HLA‐B*15:11 alleles. Vancomycin and dapsone use in HLA‐A*32:01 and HLA‐B*13:01 carriers, respectively, showed trends toward increased risk of type B ADRs.

**Conclusion:**

Utilization of pre‐stored HLA data can prevent type B ADRs including SCARs by screening high‐risk patients.

## 1 INTRODUCTION

Adverse drug reactions (ADRs) frequently occur in patients despite appropriate drug dosage and administration.^1^ Idiosyncratic type B ADRs account for approximately 20% ADRs and are mostly immune‐mediated and unpredictable.^2^ Occasionally, type B reactions can have serious consequences, such as severe cutaneous adverse reactions (SCARs) including Stevens–Johnson syndrome (SJS), toxic epidermal necrolysis (TEN), and drug‐induced hypersensitivity syndrome/drug reaction with eosinophilia and systemic symptoms (DRESS), resulting in death.^3^, ^4^ Individual genetic variability results in susceptibility to different ADRs; therefore, it is crucial to utilize the genomic data of patients for drug prescription.^3^ Currently, various genetic tests are performed in hospitals, and a vast amount of genetic information is already pre‐stored in electronic medical records (EMRs). However, this information is rarely used for indications outside its primary purpose.

Lack of integration between the genetic information of the patient and the EMRs is an obstacle in patient‐specific drug prescription at the point‐of‐care.^4^ Data on major pharmacogenomic (PGx) variants pre‐stored in the EMR should be used when prescribing high‐risk drugs to patients.^3^, ^5^, ^6^ The clinical validity of the drug–gene relationship used in this approach is mainly based on the Clinical Pharmacogenetics Implementation Consortium guidelines.^7^ Preemptive genotyping has many advantages compared to reactive genotyping. For example, the genotype information of patients can be used without delay in the prescription process. The genotype information can also be used to build a system to support physicians in making personalized prescription decisions. Furthermore, preemptive genotyping is a cost‐effective approach as many drug‐related variants can be obtained using a single panel.^8^ In reality, preemptive genotyping is not widely used in clinical practice, and PGx genes and variants found in a majority of PGx panels mainly focus on the pharmacokinetic/pharmacodynamic genes, including cytochrome P450 enzyme families.^4^ Therefore, these PGx genes are not tested for purposes other than their use in drug prescription.

In recent decades, particular human leukocyte antigen (HLA) alleles have been found to be strongly associated with the development of certain drug‐related SCARs.^9^, ^10^ We hypothesize that use of HLA PGx alleles can prevent SCARs. Despite the strong associations between some HLAs and drug‐related SCARs, pre‐stored HLA data obtained from transplant workup tests are not being utilized to screen individuals at a risk of developing SCARs when high‐risk drugs are prescribed. Storing HLA data in a structured, standardized format in EMRs is challenging as different testing methods have been used to determine HLAs over the years. Nonetheless, if pre‐existing HLA data can be successfully retrieved and re‐used based on the PGx indications, it would reduce the costs of testing and effort required to obtain the same HLA information. A clinical decision support system using the pre‐stored genetic data can also be utilized as a part of the point‐of‐care if successfully integrated.

In a previous study, we extracted, parsed, and saved the HLA data of transplant patients in a structured, standard format from pre‐stored unstructured HLA data.^11^ This study investigated the potential clinical benefits of using the extracted HLA genotypes as a risk prediction marker for ADR.

## 2 METHODS

### 2.1 Study participants

We retrospectively reviewed the medical records of patients with HLA results from January 1, 2000 to June 31, 2019, using SUPREME^®^, a clinical data warehouse of the Seoul National University Hospital (SNUH). The tested HLA gene families included HLA‐A, HLA‐B, HLA‐C, HLA‐DR, and HLA‐DQ. The study was approved by the Institutional Review Board (IRB) of Seoul National University Hospital (No. H‐1811‐157‐989).

### 2.2 Pruning human leukocyte antigen alleles and drugs associated with adverse drug reactions

**FIGURE 1:**
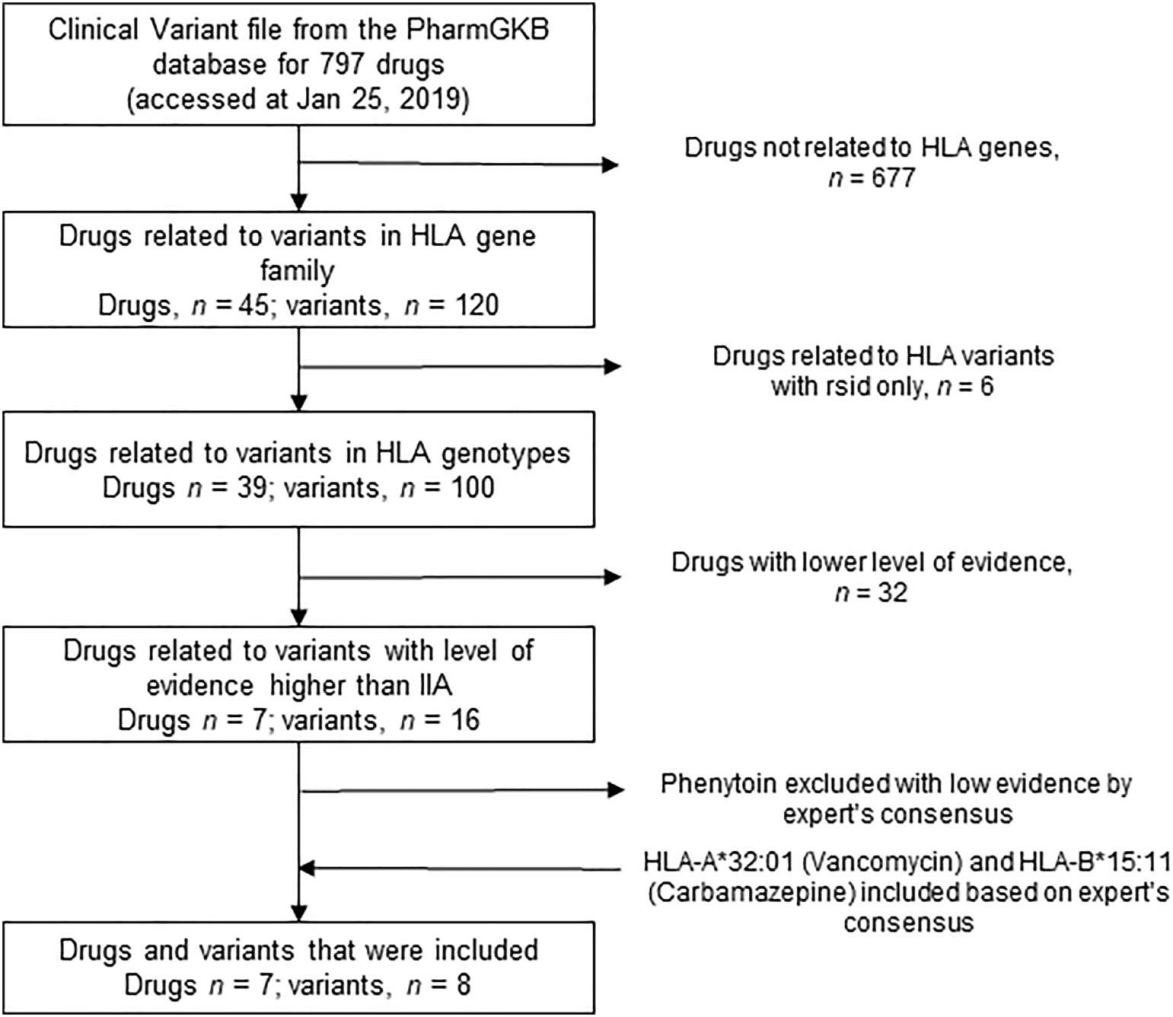
Inclusion pipeline of the ADR‐related HLA variants and drugs. ADR, adverse drug reactions; HLA, human leukocyte antigen

The gene/variant–drug relationship data were downloaded from the Pharmacogenomics Knowledge Base (PharmGKB) (accessed on January 25, 2019).^12^ Out of the 911 genes/variants and 797 drugs in the PharmGKB database, only 6 drugs and 7 HLA alleles with a high level of evidence were included in this study. Vancomycin and carbamazepine and their related HLA alleles HLA‐A*32:01 and HLA‐B*15:11, respectively, were also included after our expert panel discussion; however, they were not updated in the PharmGKB database. The detailed inclusion criteria are described in the Supplementary Materials. The following seven drugs were included: abacavir, allopurinol, carbamazepine, oxcarbazepine, dapsone, methazolamide, and vancomycin (Figure 1 **)**. We focused on eight clinically significant HLA variants, namely, HLA‐B*57:01, HLA‐B*58:01, HLA‐A*31:01, HLA‐B*15:02, HLA‐B*15:11, HLA‐B*13:01, HLA‐B*59:01, and HLA‐A*32:01, for the previously pruned drugs. As described in the Supplementary Materials, owing to the difference in the levels of representation of the HLA alleles, the HLA alleles were converted from the PharmGKB format to a 4‐digit level (Table S1). Table 1 presents the list of drugs, HLA alleles, and related ADRs included in this study. The level of evidence of the relationship in Table 1 was verified from PharmGKB, and the references were based on the PharmaGKB database or experts' review.

**TABLE 1:** List of the ADR‐related HLA genotypes and drugs

Drugs	HLA genotype	Level of evidence^a^	Adverse drug reactions	References
Abacavir	HLA‐B*57:01	1A	Drug hypersensitivity	^32^
Allopurinol	HLA‐B*58:01	1A	Drug hypersensitivity, SJS, TEN	^33^, ^34^, ^35^
Carbamazepine	HLA‐A*31:01	1A	Cutaneous ADR	^36^, ^37^, ^38^, ^39^
	HLA‐B*15:02	1A	Drug hypersensitivity, DRESS, SJS, TEN, toxic maculopapular exanthema	
	HLA‐B*15:11	‐	SJS	
Oxcarbazepine	HLA‐A*31:01	‐	DRESS	^40^, ^41^
	HLA‐B*15:02	1A	SJS	
Dapsone	HLA‐B*13:01	2A	Drug hypersensitivity, SCAR	^42^, ^43^
Methazolamide	HLA‐B*59:01	2A	SJS, TEN	^44^, ^45^, ^46^
Vancomycin	HLA‐A*32:01	‐	DRESS	^47^

Abbreviations: ADR, adverse drug reaction; DRESS, drug reaction with eosinophilia and systemic symptoms; HLA, human leukocyte antigen; SCAR, severe cutaneous adverse reactions; SJS, Stevens–Johnson syndrome; TEN, toxic epidermal necrolysis.

^a^ From PharmGKB annotation.

### 2.3 Identifying patients with human leukocyte antigen‐related adverse drug reactions

To determine the number of patients who experienced HLA‐related ADRs due to the seven drugs included in this study, the prescription data of participants, HLA allele information, diagnostic codes of type B ADRs such as “toxic maculopapular eruption,” “acute generalized exanthematous pustulosis (AGEP),” “SJS,” “TEN,” “DRESS syndrome,” and “drug eruption” were reviewed in SUPREME^®^. The ADRs that we used were classified and represented in Figure S1. We utilized two different data sources to determine the number of patients who experienced HLA‐related ADR due to the seven drugs included in this study. The first was a database of all adverse drug events, ICSR (Individual Case Safety Reports database) which is officially referred to investigate all drug side effects in inpatients of the SNUH by the Drug Safety Center since January 1, 2009. The ICSR was very confirmatory database collected by the full investigation of trained clinicians for each ADR‐suspected patient. The second one was the diagnostic code of EHR extracted from SUPREME^®^. In addition to the diagnostic code, HLA prescription information and HLA allele information were also obtained from SUPREME^®^. To target only drug side effects that are likely to be related to HLA, we first limited the types of ADRs to Type B reactions. We divided the Type B reactions into mild/moderate and severe according to the severity because it is challenging to define a causal relationship between the various ADRs and the drugs, especially when the ADRs are mild or subtle such as a slight skin rash. We classified “toxic maculopapular eruption,” “acute generalized exanthematous pustulosis (AGEP),” “SJS,” “TEN,” and “DRESS syndrome,” as severe Type B reactions, and if a patient ever had this diagnosis in EHR or had been reported in the ICSR with one of those diagnoses, this patient was defined as showing a severe Type B reaction. Considering that if the patient's ADRs are very certain and severe, the attending physician is likely to register for the diagnosis immediately, we used both diagnosis codes and ADRs report. This is because if a ADR is very clear and causal relationship is certain, the physician might not consult the investigation for ADRs to Drug Safety Center. On the other hand, in the case of mild/moderate Type B, it was difficult to confirm a causal relationship by the dianosis code with retrospective record review, so we only used the reports of the ICSR, which clearly reported a causal relationship (Table S2).

### 2.4 Statistical analysis

Statistical analysis focused on the ADR events according to the risk drug prescription and HLA genotype status. Categorical variables were compared between the two groups, with and without HLA risk alleles. A 2 × 2 table was made for each prescribed drug to compare the frequencies of patients with HLA PGx alleles and ADR occurrence. The *p*‐values, odds ratios (OR), and 95% confidence intervals (CI) were calculated using a two‐tailed Fisher's exact test. A *p*‐value less than 0.05 was considered statistically significant. Haldane's correction was used by adding one to all cells if a SCAR or type B ADR was not reported in patients without the HLA PGx alleles.^13^ Haldane's correction was not used if a SCAR or type B ADR was not reported in patients with the HLA PGx alleles. All analyses were conducted using the *R* statistical software version 3.0.2.^14^

## 3 RESULTS

### 3.1 Characteristics of human leukocyte antigen‐tested patients and allele frequency

The clinical characteristics of 11,988 patients with HLA testing are summarized in Table 2. Overall, 36.7% patients were female. The indications for HLA genotyping were as follows: 39.7% for kidney transplantation, 15.8% for bone marrow transplantation, 9.2% for liver transplantation, 1.8% for donors, 1.2% for lung transplantation, 0.4% for multiple organ transplantation, 0.3% for heart transplantation, and 0.7% for other transplantations.

**TABLE 2:** Clinical characteristics of study participants (*n* = 11,988)

Characteristic	Total (*n* = 11,988)
Age (mean/SD)	44.28 ± 17.00
Female	4402 (36.7%)
Indications for HLA genotyping test	
Kidney transplantation	4754 (39.7%)
BM transplantation	1897 (15.8%)
Liver transplantation	1104 (9.2%)
Organ donors	217 (1.8%)
Lung transplantation	139 (1.2%)
Other indications	81 (0.7%)
Multiple organ transplantation	45 (0.4%)
Heart transplantation	36 (0.3%)
Recipient candidates for undefined transplantation	3715 (31.0%)
Number of participants tested for the HLA gene	
HLA‐A	8671 (72.3%)
HLA‐B	11,929 (99.5%)
HLA‐C	4033 (33.6%)
HLA‐DRB1	8484 (70.8%)
HLA‐DQB1	2453 (20.5%)

Abbreviations: HLA, human leukocyte antigen; SD, standard deviation.

There were changes to the HLA testing methods over the 10‐year study duration, and different testing methods were used depending on the transplanted organ. In total, 11,929 (99.5%) patients underwent HLA‐B typing, whereas HLA‐DQB1 typing was performed least with the frequency of 20.5% (Table 2).

### 3.2 Combined results of human leukocyte antigen testing and prescription records

**FIGURE 2:**
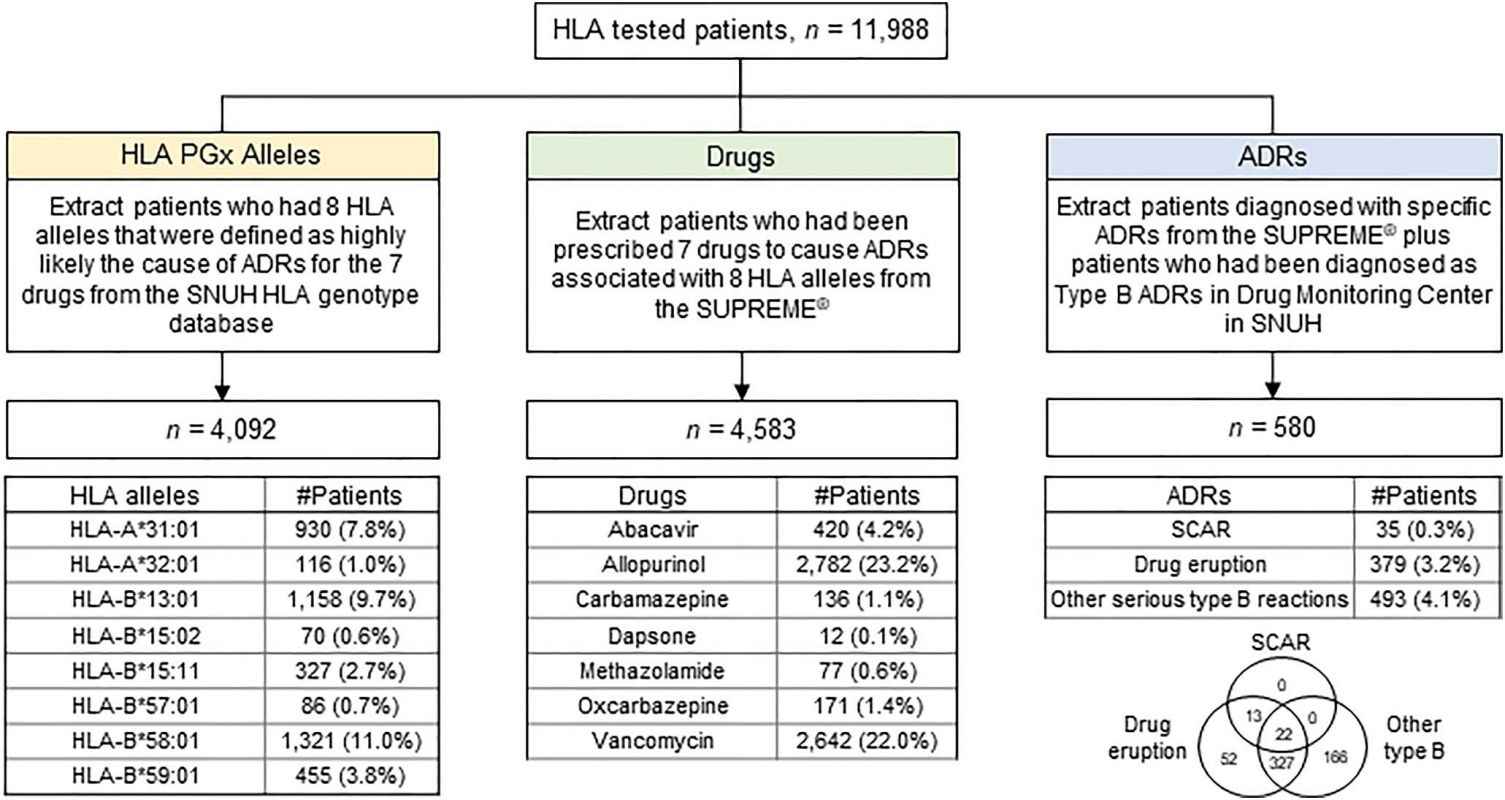
Frequency of pharmacogenomics HLA allele, drug, and adverse drug reactions in patients with pre‐stored HLA data. The left side process represents patients with HLA alleles associated with ADRs (seven alleles); the middle process represents patients who were prescribed medications (seven drugs) with known HLA‐associated ADRs; and the right side process represents HLA‐related ADRs from the Drug Safety Center reports and review of the diagnoses. HLA, human leukocyte antigen; PGx, pharmacogenomics; ADRs, adverse drug reactions; SNUH, Seoul National University Hospital; SJS, Steven–Johnson syndrome; DE, drug reaction with eosinophilia and systemic symptoms

We investigated the status of the eight ADR‐related HLA‐alleles, seven risk drugs, and target ADRs reported in 11,988 HLA‐tested patients, as shown in Figure 2. In total, 4092 patients (34.1%) had at least one of the risk HLA alleles. The HLA‐B*58:01 allele had the highest frequency at 11.0% (Figure 2). In addition, 4583 patients (38.2%) were prescribed at least one of the seven risk drugs. The drug with the highest number of prescriptions was allopurinol (*n* = 2782, 23.2%).

Some discrepancies in the reports of adverse events were identified using SUPREME^®^ and the ICSR. To resolve the discrepancies, we regarded all cases of the reported drug‐related side effects in either of the two databases (SUPREME^®^ and ICSR) as true. Additionally, when the severity of ADRs reported in the two databases did not match, we assumed that more severe events took place. Data of a total of 580 patients with ADRs related to the seven PGx drugs were evaluated.

### 3.3 Hypersensitivity risk according to human leukocyte antigen alleles

A total of 4910 patients had at least one of the eight HLA PGx alleles or were prescribed at least one of the seven PGx drugs. Of these patients, 1597 had both one of the HLA PGx alleles and a PGx drug prescription. Table 3 shows the number of patients who took the PGx drugs, those who experienced SCAR or type B ADRs, and those with HLA PGx alleles, as well as their overlapping frequencies.

**TABLE 3:** Comparison of the frequency of SCAR and Type B ADR occurrence according to patient's HLA PGx allele status

PGx drugs	PGx alleles	PGx drug(+)^a^	SCAR (+)^b^	SCAR(+)/PGx allele (+)^c^	SCAR(+)/PGx allele (−)^d^	Preventable SCAR cases^e^	Type B (+)^b^	Type B (+)/PGx allele (+)^c^	Type B (+)/PGx allele (−)^d^	Type B ADR preventable cases^e^
Abacavir	B*57:01	420	1	0/1 (0.0%)	1/419 (0.2%)	0	9	0/1 (0.0%)	9/419 (2.1%)	0
Allopurinol	B*58:01	2782	15	7/309 (2.3%)	8/2473 (0.3%)	7 (46.7%)	347	53/309 (17.2%)	294/2473 (11.9%)	53 (15.3%)
Carbamazepine	A*31:01	136	1	1/11 (9.1%)	0/125 (0.0%)	1 (100%)	9	3/11 (27.3%)	6/125 (4.8%)	3 (33.3%)
	B*15:02									
	B*15:11									
Oxcarbazepine	A*31:01	171	2	1/15 (6.7%)	1/156 (0.6%)	1 (50%)	17	4/15 (26.7%)	13/156 (8.3%)	4 (23.5%)
	B*15:02									
Methazolamide	B*59:01	77	0	0/4 (0.0%)	0/73 (0.0%)	0	1	0/4 (0.0%)	1/73 (1.4%)	0
Dapsone	B*13:01	12	2	2/3 (66.7%)	0/9 (0.0%)	2 (100%)	3	2/3 (66.7%)	1/9 (11.1%)	2 (66.7%)
Vancomycin	A*32:01	2642	18	0/39 (0.0%)	18/2603 (0.7%)	0	341	9/39 (23.1%)	332/2603 (12.8%)	9 (2.6%)

Abbreviations: ADR, adverse drug reaction; PGx, pharmacogenomics; SCAR, severe cutaneous adverse reaction; Type B, Type B adverse reaction.

^a^ These numbers represent the patients who were prescribed the PGx drug.

^b^ These numbers represent the patients who had SCAR or Type B ADR among the patients who were prescribed the drug with/without HLA PGx alleles.

^c^ These numbers represent the patients who had SCAR or Type B ADR among the patients who were prescribed the drug with the HLA PGx alleles.

^d^ These numbers represent the patients who had SCAR or Type B ADR among the patients who were prescribed the drug without the HLA PGx alleles.

^e^ These numbers represent the patients with the HLA PGx alleles among the patients who had SCAR or Type B ADR.

No case was reported with a SCAR or type B ADR among patients who took abacavir and also had the HLA‐B*57:01 allele. In case of allopurinol, 2782 patients had been prescribed the drug, and 1321 patients had the HLA‐B*58:01 allele. We identified 309 patients with the HLA‐B*58:01 allele who had been prescribed allopurinol. Of these patients, 7 (2.3%) developed a SCAR and 53 (17.2%) developed type B ADRs, as shown in Table 3. The OR of developing SCAR in allopurinol‐prescribed patients with the HLA‐B*58:01 allele was 7.13 (95% CI 2.19–22.69, *p* < 0.0001). Idiosyncratic type B ADRs, including SCAR, also showed a significant difference (*p* = 0.001) with an OR of 1.53 (95% CI 1.09–2.13) as shown in Table 4.

**TABLE 4:** Comparison of risk of side effects depending on whether or not the drug was prescribed in patients with risk allele

Drug	HLA allele	Type of ADR	Odds ratio (95% CI)	*p‐*value
Allopurinol	B*58:01	SCAR	7.13 (2.19–22.69)	<0.0001
		Type B ADR	1.53 (1.09–2.13)	0.001
Carbamazepine	A*31:01	SCAR	21.72 (1.05–1346.71)	0.023
	B*15:02	Type B ADR	7.22 (0.99–42.64)	0.026
	B*15:11			
Oxcarbazepine	A*31:01	SCAR	10.73 (0.13–867.28)	0.168
	B*15:02	Type B ADR	3.95 (0.80–16.01)	0.046
Dapsone	B*13:01	SCAR	11.81 (0.68–856.20)	0.063
		Type B ADR	11.09 (0.34–1044.70)	0.127
Vancomycin	A*32:01	SCAR	NA	NA
		Type B ADR	2.05 (0.85–4.48)	0.086

Abbreviations: ADR, adverse drug reaction; CI, confidence interval; HLA, human leukocyte antigen; inf, infinite; SCAR, severe cutaneous adverse reaction.

**FIGURE 3:**
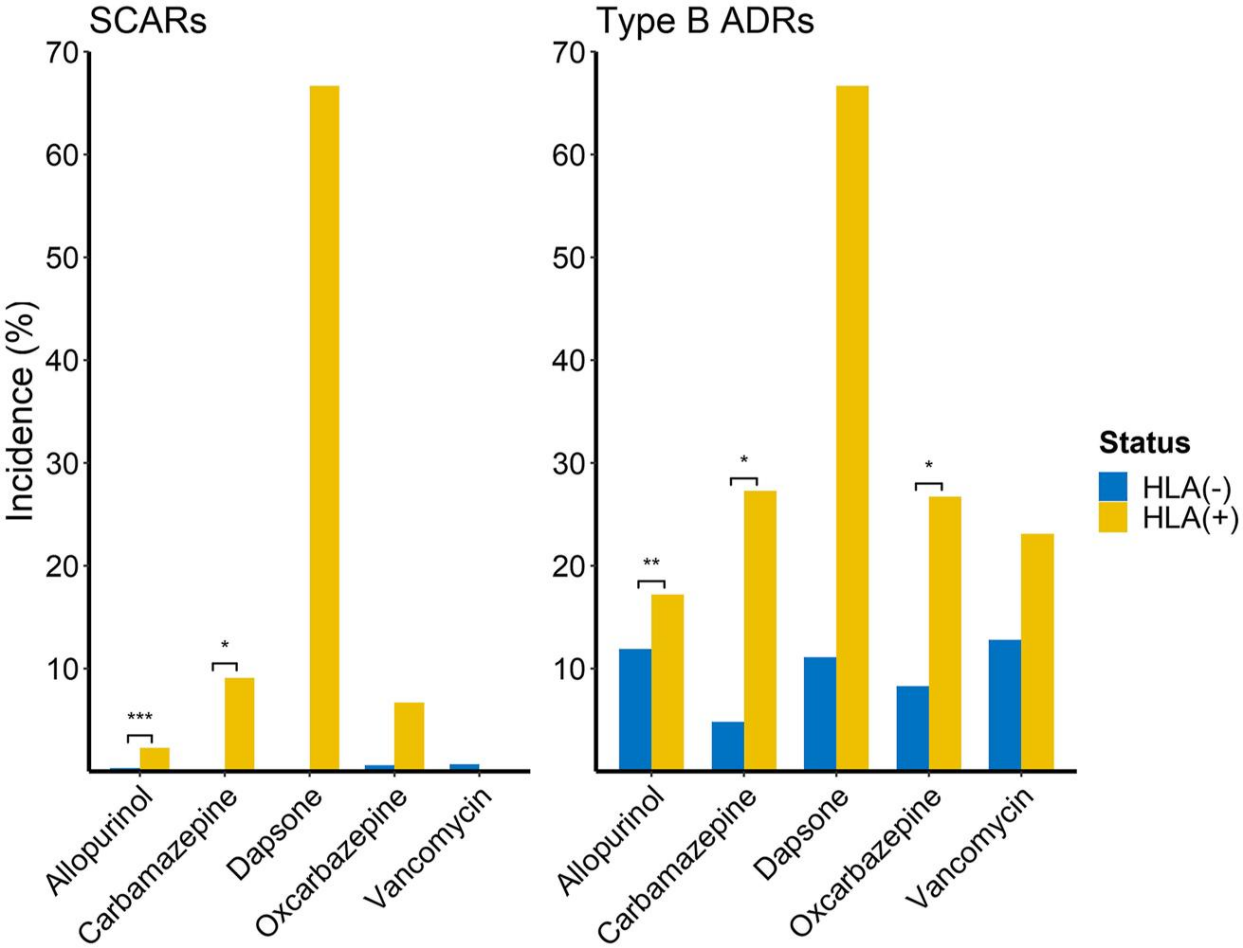
Comparison of the frequency of type B ADR and SCAR incidence for allopurinol, carbamazepine, oxcarbazepine, and vancomycin. ADR, adverse drug reactions; HLA, human leukocyte antigen; SCAR, severe cutaneous adverse reaction

For carbamazepine and oxcarbazepine administration, there was only one patient who had been diagnosed with a SCAR. This patient had the HLA‐A*31 allele. Because there were no SCAR patients in the group of patients without the three risk alleles HLA‐A*31:01, HLA‐B*15:02, and HLA‐B*15:11 who were prescribed carbamazepine, Haldane's correction was used. After the correction, the OR of SCAR occurrence in the carbamazepine group with one of the three risk alleles was 21.72 (95% CI 1.05–1346.71, *p* = 0.023). The difference in type B ADR occurrence for carbamazepine was also statistically significant as 27.3% (3/11) of patients with the risk alleles and 4.8% (6/125) of patients without the risk alleles developed type B ADRs, and the OR was 7.22 (95% CI 0.99–42.64, *p* = 0.026). The data on oxcarbazepine (related to HLA‐A*31:01 and HLA‐B*15:02) administration also showed some increase in the number of patients with risk alleles. The indications for carbamazepine and oxcarbazepine were typically the same, and HLA‐B*15:11 was the only difference in the reported risk alleles between the two; therefore, the data for these two drugs were combined and analyzed. The OR for SCAR was 9.0, which was not statistically significant (95% CI 0.11–714.8, *p* = 0.19); however, for type B ADR, the OR was 4.15 (95% CI 1.32–11.74, *p* = 0.007), which showed a significant difference. Among the 77 methazolamide users, there was one case of type B ADR but no one had a SCAR. Among the 12 dapsone users, 2 of 3 (66.7%) patients with the HLA‐B*13:01 allele had SCARs, whereas no patients (0%) were reported with a SCAR among those without any risk alleles. Although we identified a large difference in the SCAR occurrences between the two groups (with and without risk alleles), this was not statistically significant owing to the small sample size of dapsone users. For vancomycin, a type B ADR due to vancomycin occurred in 23.1% patients with HLA‐A*32:01 and 12.8% patients without HLA‐A*32:01. The OR for the development of type B ADR in patients with the risk allele was 2.05 (95% CI 0.85–4.48, *p* = 0.086); however, it was not statistically significant. There were no cases with vancomycin‐related SCARs among nine patients with the HLA‐A*32:01 allele. Overall, the risk of developing SCARs due to allopurinol and carbamazepine use and type B ADRs due to allopurinol, carbamazepine, and oxcarbazepine use were significantly higher in patients with the risk alleles (Figure 3).

It is assumed that if patients with the risk alleles had not been prescribed the high‐risk drugs, the following SCARs would have been prevented: 7/15 (46.7%) for allopurinol, 1/1 (100%) for carbamazepine, 1/2 (50%) for oxcarbazepine, and 2/2 (100%) for dapsone (Table 3).

## 4 DISCUSSION

Our study showed for the first time the simulated benefits of utilizing pre‐stored HLA information to prevent type B ADRs or SCARs without additional testing in a real‐life setting. Consistent with the findings of previous studies, the risk of developing SCARs due to allopurinol and carbamazepine use was significantly higher in patients with the risk alleles. In addition, type B ADRs due to allopurinol, carbamazepine, and oxcarbazepine use were more common in patients with the risk alleles. Conversely, if the data on the HLA PGx alleles were available before prescribing the risk drugs to the patients, a significant number of SCARs and type B ADRs could have been prevented, resulting in improved patient safety and cost effectiveness.

Our findings support the claim that PGx information should be integrated into EMRs using a clinical decision support system.^5^, ^15^ In case of organ transplantation patients who have already undergone tests for HLA genotyping, physicians could use this HLA PGx information to reduce ADR risks. Although our study did not find statistically significant differences in the rate of ADRs for some drugs, possibly owing to the small number of the study population who took them, the available HLA data could be useful in preventing ADRs.

In addition to the data on the HLA PGx alleles, other readily available HLA allele data can be used to diagnose and identify individuals at a risk of various autoimmune diseases.^16^, ^17^ Associations between certain HLA alleles and autoimmune diseases, such as HLA‐DRB1 for rheumatoid arthritis, *HLA‐B51* for Behcet's disease, *HLA‐B27* for ankylosing spondylitis, HLA‐DQ2/DQ8 for celiac disease, and HLA‐DQB1*06:02 for narcolepsy, are widely known. A broadened screening of pre‐stored HLA information to detect disease‐associated variants of HLA alleles may enable early identification of susceptible patients, which may facilitate early diagnosis of diseases.

The exact incidence of SCARs is unknown; however, the incidence of SJS/TEN is estimated at 1–2 cases per 1,000,000 people per year.^18^ Although the incidence of SCARs is very low, it carries significant morbidity, and the mortality rates are 10% for SJS, 30% for SJS/TEN overlap, 50% for TEN, and 5% for DRESS.^18^ Furthermore, SCARs may severely damage the affected mucosa or skin and leave permanent sequelae. Therefore, although the absolute risk reduction is small owing to its rare occurrence, the potential benefits of preventing SCARs are substantial. Considering patient data for the HLA PGx alleles is already available, it is reasonable to integrate this data and ensure its availability for clinicians at the point‐of‐care to help prevent SCARs. To achieve this, a clinical decision support system that instantly informs the personalized estimated risk to physicians by automatically linking the existing genetic information with the prescription should be implemented.

Pharmacogenomic studies showed that certain HLA genotypes induce T cell activation to a specific drug, resulting in the development of a SCAR. In 2005, the HLA‐B*58:01 allele was first reported to be strongly associated with allopurinol‐induced SCAR in a case–control study of the Han Chinese population in Taiwan (OR = 580.3).^19^ In Korea, the HLA‐B*58:01 allele was also strongly associated with allopurinol‐induced SCAR (OR = 97.8).^20^ This study is consistent with these findings, confirming the usefulness of detecting the HLA‐B*58:01 allele in actual clinical practice.

However, the association between a specific allele and a particular drug‐induced hypersensitivity varies between ethnicities. For example, 100% of carbamazepine‐induced SJS patients were positive for the HLA‐B*15:02 allele, whereas only 3% of the tolerant patients were positive (OR = 2504) in the Han Chinese population, in which the HLA‐B*15:02 allele frequency is relatively high.^21^ This strong association between the occurrence of carbamazepine‐induced SJS/TEN and HLA‐B*15:02 was not replicated in Koreans, in whom the HLA‐B*15:02 allele frequency is low.^22^ Instead, HLA‐B*15:11 has been proposed as an additional allele type associated with carbamazepine‐induced SJS in Koreans.^23^ In patients with carbamazepine‐induced DRESS, the HLA‐A*31:01 allele was reported as a risk marker in Europeans, Japanese, and Koreans.^23^ Oxcarbazepine, a 10‐keto analog of carbamazepine, has also been associated with the HLA‐B*15:02 and HLA‐A*31:01 alleles in the development of SJS/TEN and maculopapular rash, respectively.^24^

Methazolamide, a carbonic anhydrase inhibitor used as an intraocular pressure‐lowering drug to treat glaucoma, was found to be associated with SJS/TEN in individuals with the HLA‐B*59:01 allele in Northeast Asia, including Korea, Japan, and China.^25^ However, we did not find significant results regarding methazolamide because its use was minimal in our study. Dapsone, an antimicrobial agent used in the treatment of leprosy or *Pneumocystis jirovecii* pneumonia, can induce a hypersensitivity syndrome similar to DRESS syndrome,^26^ and it was significantly associated with the HLA‐B*13:01 allele in Chinese, Thai, and Koreans.^27^ Recently, the HLA‐A*32:01 allele was reported to be strongly associated with vancomycin‐induced DRESS. Although vancomycin is one of the main culprit drugs of SCARs in Korea, the HLA‐A*32:01 allele is unlikely to be used as a screening test considering its rare genotype frequency in Koreans, which is at 0.6%.^27^ Further studies to investigate the risk alleles of vancomycin‐induced DRESS in the Korean population are warranted. Nonetheless, if pre‐stored data on HLA‐A*32:01 were readily available, it could still be used to minimize vancomycin prescription in susceptible patients with the HLA‐A*32:01 allele.

Abacavir, a nucleoside analog used to treat HIV infections, can cause severe delayed systemic hypersensitivity reactions in association with the HLA‐B*57:01 allele. However, the use of the HLA‐B*57:01 allele as a screening test has no clinical relevance owing to its very low allelic frequency (0.2%) in Koreans.^28^ Our results are consistent with this observation.

Of the 4092 individuals with 8 HLA PGx alleles identified in our study, 1597 (39%), or 13% of the total number of study subjects, were prescribed one or more of the seven drugs associated with these alleles. If all these patients had their HLA test results evaluated at the time of drug prescription, doctors could have made better therapeutic decisions, including whether to prescribe alternative drugs, change the dose, or carefully monitor the patients according to their individual PGx profile. In addition, if a prescription was changed by a physician due to an automatic PGx warning at the time of prescription, not only SCARs but also a considerable number of type B ADRs, specifically 15.3% (53/347) for allopurinol, 33.3% (3/9) for carbamazepine, 23.5% (4/17) for oxcarbazepine, 66.7% (2/3) for dapsone, and 2.6% (9/341) for vancomycin, could have been prevented.

Our study is a retrospective study and the preventative effects proposed need to be assessed further in prospective studies and real clinical practice. Nonetheless, the proposed approach has clear benefits because it utilizes pre‐tested HLA data to prevent ADR occurrences without additional costs or patient discomfort. Given the rapid increase in the genomic data collection for research and clinical purposes, timely use of these valuable genomic data in clinical practice should be prioritized.^29^, ^30^ For the secondary use of the point‐of‐care of genomic tests, it is necessary to evaluate and confirm the clinical validity of the tests for the specific uses other than the primary purpose. Additionally, it is necessary to devise an effective and safe approach to deliver genomic information at the right time and in the right manner. The most appropriate method so far is to design and use a clinical decision support system that integrates the personal genomic information of the patient at the time of drug prescription and provides an appropriate PGx warning. Considering the diversity of genetic tests, various drugs, and individual genetic variations, it is necessary to design an optimal system to ensure efficiency and safety in clinical practice. Based on Table 3, if the HLA genotyping test was used for screening, the positive predictive value of SCAR occurrence by the HLA‐B*58:01 allopurinol drug was 0.32%, and the negative predictive value was 97.73%. This suggests that the HLA test can be used to predict in advance whether a patient is likely to develop SCAR when using a risk drug with known HLA‐related SCAR occurrence.

Although this study provided important results, there are several limitations. First, the HLA genotypes selected in this study were obtained from results accumulated over a long period using various HLA testing methods. Therefore, the results of the lower resolution HLA tests were converted to the matched 4‐digit results based on the previously reported population‐wise allele frequencies. This means that some results may not be accurate in some patients. However, this is unlikely to result in significant error based on the allele frequency data.^28^ For example, the *HLA‐B58* status was also shown to be strongly associated with allopurinol hypersensitivity and HLA‐B*58:01 is the only *HLA‐B58* allele in the Korean population.^31^ Second, the causal relationships in this study were not assessed by confirmative tests, and therefore, misdiagnosis and overestimation are possible. In our analysis, we assumed that the reported SCARs or type B ADRs were related to the prescribed drug. Therefore, SCARs reported in an individual could have been caused by another drug other than the risk drug associated with the HLA PGx alleles. The study relied on ADR reporting, and thus, under‐reporting could have been a problem. Nonetheless, our study could replicate the significant associations between the HLA‐B*58:01 allele and allopurinol and the HLA PGx alleles for carbamazepine, suggesting that our overall findings are valid. Third, we obtained whether a patient experienced drug side effects from two sources: the diagnosis name of CDW and the Drug Safety Center side effect reporting database. In the case of severe Type B ADRs (AGEP, SCAR, DRESS), patients reported at either site were classified as having serious Type B ADRs. This approach might cause some overestimation for patients with adverse events. However, whether the patient experienced ADRs and whether the HLA test was performed were independent events, so it would not have affected the frequency of Type B ADRs according to the presence of the HLA allele.

In summary, the results of this study highlight the importance of utilizing pre‐stored HLA data to predict potential ADRs in a clinical setting. Because HLA PGx alleles are already available, no additional cost is required; moreover, if these HLA PGx alleles were readily available at the point‐of‐care, some SCARs and type B ADRs could have been prevented. Therefore, further studies are needed to integrate HLA data and existing EMRs to achieve a personalized medicine approach.

## CONFLICT OF INTERESTS

The authors have declared no conflicts of interest.

## Supporting information

Supporting Information S1
