# Supplementary material for: Reducing severe cutaneous adverse and type B adverse drug reactions using pre‐stored human leukocyte antigen genotypes
Source: Clin Transl Allergy. 2022 Jan 14;12(1):e12098. doi: 10.1002/clt2.12098 (PMC8760506; doi:10.1002/clt2.12098)
Supplement: Supplementary file 1 — Supporting Information S1 [file CLT2-12-e12098-s001.docx]

**Supplementary materials**

**Supplementary Methods**

**Process to exclude drugs/alleles with low level of evidence**

To gather data for which ADRs associated with HLA genotypes were reported, we first downloaded clinical annotation data from Pharmacogenomics Knowledgebase (PharmGKB) (accessed on Jan 25, 2019). The downloaded clinicalvariant.tsv file contained 911 genes and variants known to be involved in unexpected drug responses, and included data related to pharmacokinetics (dosage, metabolism) and pharmacodynamics (efficacy), and toxicity/ADR for 797 drugs. Excluding genes had a relationship other than HLA genes or rsid only, there were 39 drugs and 100 variants included. In order to target only the clinically relevant mutations related to the action and adverse reactions of the drug, we included only those drugs corresponding to the evidence level IIA or higher of PharmGKB and the drugs-related thereto. These included 7 drugs and 16 mutations. Even for drugs which reported to have high relevance as per that PharmGKB, a review by two experts in allergy and pharmacogenomics was done so as to select only those mutations that were fully recognized through actual linked research and clinical experience. Through this expert review, we focused the level of evidence and ethnic specificity of HLA-related ADRs for specific HLA variants and the related drugs. Since HLA-related ADRs are known to differ between ethnicities, we reviewed a paper on the ADRs of drug-related to HLA allele in Korea, and the results were further reviewed by experts. Based on this review, nevirapine was excluded because it had not been reported to cause ADRs based on HLA genotypes in the Korean/East Asian population. Two other drugs, an antithyroid drugs and aspirin, which were related to HLA genotype of HLA-B*38:02:01 and HLA-DPB1*03:01:01 from the PharmGKB, were excluded based on the decision of the expert panel due to their low pathogenicity and low evidence level. In addition, phenytoin was excluded and vancomycin (HLA-A*32:01) was included. The HLA-B*15:11 for carbamazepine also was added to the analysis target variants. As a result, we targeted eight HLA genotypes for seven drugs.

**Matching level of resolution for the HLA allele representation**

We focused on the eight clinically important HLA variants: HLA-B*57:01, HLB-B*58:01, HLA-A*31:01, HLA-B*15:02, HLA-B*15:11, HLA-B*13:01, HLA-B*59:01, and HLA-A*32:01 for the seven previously pruned drugs. The description of these variations was done by HLA nomenclature. Except for the two mutations, HLA-B*58:01, HLA-A*32:01, the other mutations were marked with six-digit length, representing specific HLA protein separated by colon after every two digits, according to the standard HLA nomenclature. However, in many cases, our patients' HLA test results were described only to serotyping (two-digit length, which represents HLA allele group), depending on the test method (Supplementary File 1.xlsx). In addition, most of them indicated only specific HLA protein of four-digit length. Since the phenotype may vary depending on whether the HLA coding sequence is a synonymous variant (six-digit length) or a non-coding variant (eight-digit length), we confirmed the HLA allele frequency through literature review. If the specific HLA protein reported in a population has 100% a nonsynonymous variant, the high-resolution variants type (six-digit length), as suggested by PharmGKB, could be converted to protein type (four-digit length) or even serotype (two-digit length), and mapped. Through literature search and expert panel discussion, we confirmed that there were no issues with the eight variants evaluated in this study (Table 1), which had a four-digit length (excluding the last two digits in the case of a six-digit length description), and increasing the resolution.

In cases when only the serotype of the patient was confirmed (according to the frequency of HLA alleles known in Korea), it was classified assuming that if the specific allele type in the serotype is 95% or more, the allele type. For example, HLA-B*57 serotype is known as allele type HLA-B*57: 01 in 95.8% (HLA-B*57: 01: 0.23%, HLA-B*57: 02: 0.01%), allotype HLA-B*57:01 was classified for patients identified as serotype HLA-B*57. Likewise, for HLA-A*31 serotype, allele type HLA-A*31:01 (99.6%), for HLA-A*32 serotype, allele type HLA-A*32:01 (98.3%), HLA-A*33 serotype was classified as allele type HLA-A*33:03 (99.7%). Table 1 presents the list of drugs finally included in the analysis, the HLA alleles, and related ADRs. The level of evidence of the relationship were verified from the PharmGKB and the reference were based on the data available in PharmaGKB and expert review.


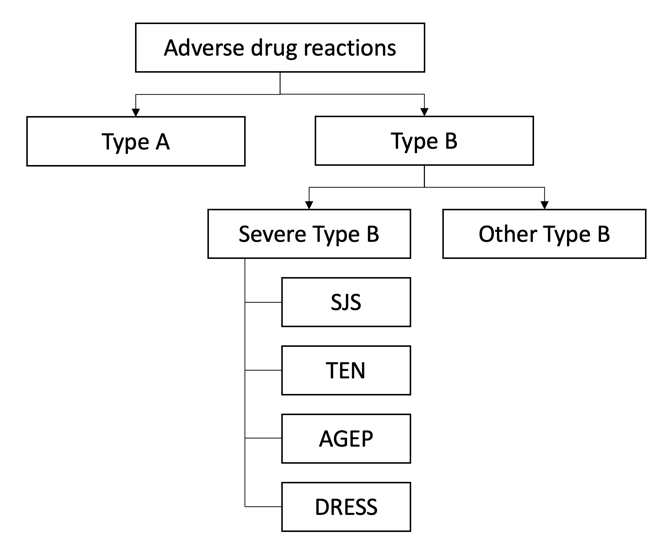


**Supplementary Figure 1.** HLA-related Type B adverse drug reaction extraction process

**Supplementary Table 1.**


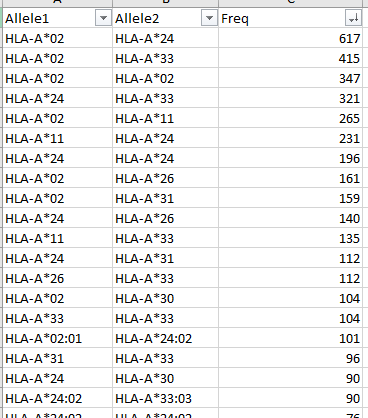


**Supplementary Table 2. Allele frequency of PGx HLA genes among patients from the SNUH HLA genotype database**

| No | Drugs | HLA genotype from PharmGKB [A] | #Patients with A | Shortened HLA allele annotation [B] | #Patients with B | Changed annotation of SNUH HLA database [C] | #Patients with B after processing of C |
| --- | --- | --- | --- | --- | --- | --- | --- |
| 1 | abacavir | HLA-B*57:01:01 | 0 | HLA-B*57:01 | 21 | HLA-B*57 -> HLA-B*57:01 | 86 |
| 2 | allopurinol | HLA-B*58:01 | 1236 | HLA-B*58:01 | 1236 | HLA-B*58 -> HLA-B*58:01 | 1320 |
| 3 | cabamazepine | HLA-A*31:01:02 | 0 | HLA-A*31:01 | 161 | HLA-A*31 -> HLA-A*31:01 | 930 |
|  |  | HLA-B*15:02:01 | 0 | HLA-B*15:02 | 70 | . | 70 |
| 4 | dapsone | HLA-B*13:01:01 | 0 | HLA-B*13:01 | 91 | HLA-B*13 -> HLA-B*13:01 | 1158 |
| 5 | methazolamide | HLA-B*59:01:01:01 | 0 | HLA-B*59:01 | 118 | HLA-B*59 -> HLA-B*59:01 | 455 |
| 6 | oxcarbazepine | HLA-B*15:02:01 | 0 | HLA-B*15:02 | 70 | . | 70 |
| 7 | vancomycin | HLA-A*32:01 | 15 | HLA-A*32:01 | 15 | HLA-B*32 -> HLA-B*32:01 | 116 |

**Supplementary Table 3. Number of patients who diagnosed for the HLA-related ADRs queried by SUPREME**

| No | Diagnosis | Hospital Codes | #Cases | #Patients |
| --- | --- | --- | --- | --- |
| 1 | Toxic maculopapular eruption | D00008806 | 0 | 0 |
|  |  | 20002963 |  |  |
|  |  | 89045 |  |  |
| 2 | Drug eruption | D00012297 | 1583 | 107 |
|  |  | D00012299 |  |  |
|  |  | 83206 |  |  |
|  |  | 84964 |  |  |
|  |  | 83208 |  |  |
|  |  | 84965 |  |  |
| 3 | Acute generalized exanthematous pustulosis | D00012296 | 4 | 2 |
|  |  | 90612 |  |  |
| 4 | Stevens-Johnson syndrome | D00012394 | 339 | 14 |
|  |  | 75340 |  |  |
|  |  | 78308 |  |  |
|  |  | 83299 |  |  |
|  |  | 83777 |  |  |
| 5 | Toxic epidermal necrolysis | D00012395 | 7 | 6 |
|  |  | 83209 |  |  |
|  |  | 84966 |  |  |
| 6 | DRESS | D00020951 | 88 | 15 |
|  |  | 20004081 |  |  |
| Total | | | 2021 | 125 |
